# Analysis of peripheral doses for base of tongue treatment by linear accelerator and helical TomoTherapy IMRT

**DOI:** 10.1120/jacmp.v11i3.3136

**Published:** 2010-06-21

**Authors:** Brian Richard Bennett, Michael A. S. Lamba, Howard R. Elson

**Affiliations:** ^1^ The Barrett Cancer Center University of Cincinnati Cincinnati Ohio 45267 USA

**Keywords:** intensity‐modulated radiation therapy, peripheral dose, whole‐body dose, radiation dosimetry, risk

## Abstract

The purpose of this study was to compare the peripheral doses to various organs from a typical head and neck intensity‐modulated radiation therapy (IMRT) treatment delivered by linear accelerator (linac) and helical TomoTherapy.

Multiple human CT data sets were used to segment critical structures and organs at risk, fused and adjusted to an anthropomorphic phantom. Eighteen contours were designated for thermoluminescent dosimeter (TLD) placement. Following the RTOG IMRT Protocol 0522, treatment of the primary tumor and involved nodes (PTV70) and subclinical disease sites (PTV56) was planned utilizing IMRT to 70 Gy and 56 Gy. Clinically acceptable treatment plans were produced for linac and TomoTherapy treatments. TLDs were placed and each treatment plan was delivered to the anthropomorphic phantom four times.

Within 2.5 cm (one helical TomoTherapy field width) superior and inferior to the field edges, normal tissue doses were on average 45% lower using linear accelerator. Beyond 2.5 cm, the helical TomoTherapy normal tissue dose was an average of 52% lower. The majority of points proved to be statistically different using the Student's t‐test with p<0.05. Using one method of calculation, probability of a secondary malignancy was 5.88% for the linear accelerator and 4.08% for helical TomoTherapy.

Helical TomoTherapy delivers more dose than a linac immediately above and below the treatment field, contributing to the higher peripheral doses adjacent to the field. At distances beyond one field width (where leakage is dominant), helical TomoTherapy doses are lower than linear accelerator doses.

PACS number: 87.50.cm Dosimetry/exposure assessment

## I. INTRODUCTION

The goal of radiation therapy is to deliver tumoricidal doses while sparing normal tissue to the maximum extent possible. Intensity‐modulated radiation therapy (IMRT) utilizes inverse planning along with PTV objectives and organs‐at‐risk constraints to achieve optimized treatment plans. While treatment planning systems work well to calculate treatment dose in high‐dose regions, they lack the ability to accurately model the doses to the periphery well beyond the extent of the field. These regions reflect less than 5% of the prescribed dose. The inability to accurately calculate the low doses received by large volumes of normal tissue presents problems when considering long‐term sequelae of radiotherapy, such as induction of secondary malignancy.

This study assessed the peripheral doses from two IMRT modalities, linear accelerator and Hi·Art (TomoTherapy, Inc., Madison, WI) helical TomoTherapy, using a limited‐organ anthropomorphic phantom. Several papers have been published on peripheral doses, some utilizing slabs of solid water arranged in human shapes, such as in the papers by Stern(1) and Ramsey et al.(2) and some more recent studies using an anthropomorphic phantom. In Stern's study, peripheral doses that were studied resulted from the MLCs being either extended to the field edge or retracted; additionally, clinical plans were not used. Diodes were place 2 cm to 40 cm from the field edge. The results show that when the MLCs are extended, they produce lower peripheral doses. In the paper by Ramsey, peripheral doses were measured in a water‐equivalent phantom. The phantom consisted of three sets of water‐equivalent material arranged to resemble a human. Measurements were made using a cylindrical ion chamber at distances 10 cm to 30 cm from the field edges.

As demonstrated by Kase et al.,^(^ ^3^ ^)^ leakage is the primary contributor to those peripheral doses at distances greater than 20 cm, while scattered radiation is dominant at distances less than 20 cm. In linear accelerator step‐and‐shoot IMRT, the multileaf collimator (MLC) uses leaf patterns (beam segments) to shape the field. The beam will temporarily halt as the leaves move to their next position if step‐and‐shoot IMRT is performed. Helical TomoTherapy is continuous 360° rotation IMRT. Consisting of a linear accelerator mounted on continuously rotating gantry, the source rotates around the patient as the table moves, creating a helical delivery. During delivery, the beam is on the entire time while the leaves form thousands of beam segments. Given the same dose, a helical TomoTherapy treatment should deliver higher peripheral doses than a linear accelerator treatment due, largely, from the longer beam‐on times required of the unit (all other things being equal). This was expressed in a study by Mutic et al.,^(^ ^4^ ^)^ where measured peripheral doses from a head and neck treatment showed axial, linear accelerated‐based tomotherapy (Peacock/MIMiC, NOMOS, Inc., Sewickley, PA) peripheral doses to be higher. Measurements were made using thermoluminescent dosimeters (TLDs) in a water equivalent plastic phantom. The doses did show a strong dependence on the distance from the field edge, similar to Ramsey.(2) At 10 cm from the field, doses were 2.5% of the total dose, but were reduced to only 0.5% of the total dose at 30 cm. In a paper by Followill et al.,(5) a comparison of three modalities was made: conventional linear accelerator, conventional linear accelerator IMRT and linac‐based tomotherapy IMRT (Peacock/MIMiC, NOMOS, Inc., Sewickley, PA). Each modality treated the pelvis to 70 Gy. This paper showed that linac‐based tomotherapy treatments produced higher whole‐body doses and greater risk for secondary fatal malignancy. Probabilities of a secondary cancer ranged from 1–8.4% for linear accelerator based treatment and 2.8–24.4% for TomoTherapy.

By contrast, Ramsey^(^ ^2^ ^)^ reported peripheral doses from helical TomoTherapy to be lower, suggesting that even though the beam‐on times are long, the helical TomoTherapy unit produced less leakage. Reduced leakage would decrease dose beyond one field width of the treatment field, where scatter and other factors are of reduced significance. Dosimetric measurements from this study demonstrate a strong dependence of dose on distance from the field edge.

## II. MATERIALS AND METHODS

Accurate regions of interest (ROIs) were generated in an anthropomorphic phantom and were utilized to develop a clinically acceptable treatment plan for both linac and TomoTherapy. The anthropomorphic phantom, containing lithium fluoride (LiF) TLD‐100s (Thermo Fisher Scientific, Santa Fe, MN) was irradiated using each plan. The TLD measurements of the peripheral doses from each modality were used to assess the whole body dose from each treatment plan.

**Figure 1:**
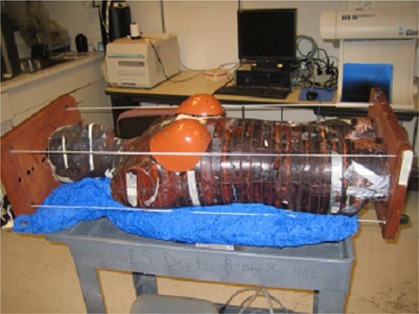
Assembled anthropomorphic phantom.

**Figure 2:**
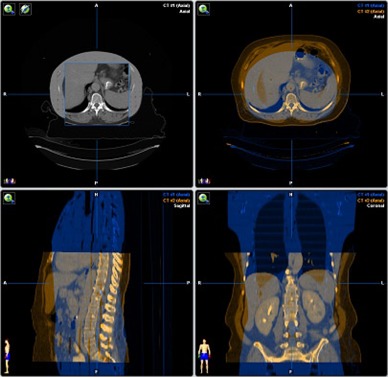
Human CT data fused with phantom CT data.

**Figure 3:**
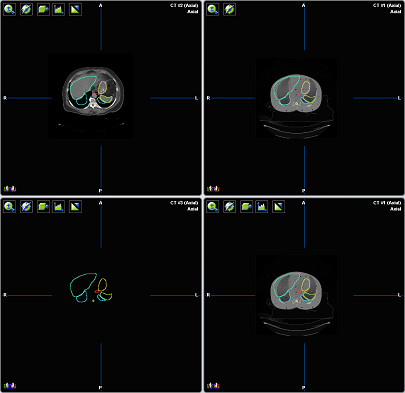
Human CT segmentations transferred to phantom data. The top left is the human dataset with contours and the top right is the phantom with contours.

**Figure 4:**
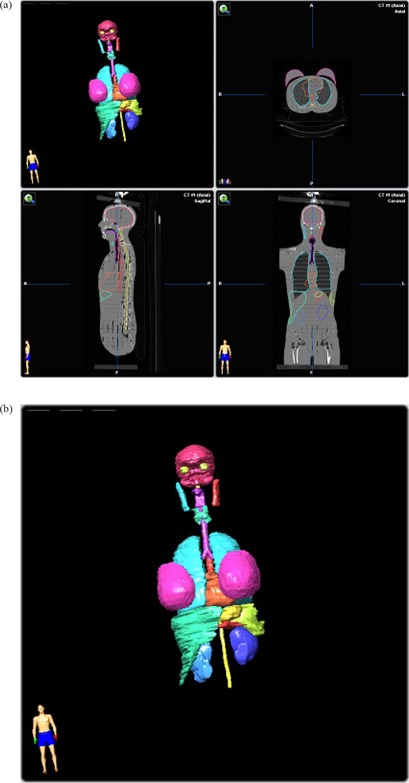
(a) Phantom CT data with contours; (b) Phantom contours.

The Alderson Rando Phantom (Radiology Support Devices, Long Beach, CA) with breast attachments – an anthropomorphic tissue‐equivalent phantom consisting of transverse sections – was assembled as in Fig. 1. The phantom was scanned and 219 3 mm slices were acquired and transferred to a fusion and contouring station. While the Rando phantom is radiologically anthropomorphic in that it has bone, lung and general soft tissue, there are no organs apparent on CT except for bone and lung. In order to generate clinically‐relevant ROI shapes, volumes, and positions, data from three different human CTs were used. Figure 2 shows the fusion of one such human CT to the phantom CT. The human ROIs were contoured, then fused in the appropriate region to the phantom, and adjusted to fit the phantom's size and shape. CTs of human head and neck, thorax, abdomen and pelvis were chosen to closely match the Rando phantom's skeleton. Figure 3 shows a contoured human CT data set that is fused to the phantom dataset. The ROIs and their volumes are listed in Table 1 and displayed in Figs. 4(a) and 4(b).

**Table 1:** Phantom organ contours and volumes.

*Organ*	*Total Volume (cm^3)*
Brain	1245.2
Brainstem	12.5
Lenses of Eyes	37.3
Parotids	59.1
Submandibular Glands	16.8
Thyroid	30.0
Breasts	844.3
Lung	4393.1
Heart	987.1
Stomach	135.3
Spleen	228.1
Liver	1189.6
Spinal Chord	113.1
Kidneys	468.6
Ovaries	6.2
Bladder	158.7

Eighteen sites were designated for point measurements on the CT dataset. The points were identified on the Rando phantom CT dataset and confirmed using radio‐opaque markers and fluoroscopy. The distances from the field edges were accurately determined using the contouring software. The “ruler” function was used to measure the distances from the nearest field edge, whether superior or inferior, to the center of the measurement site. Each site, and its respective distance from the field edge, is listed in Table 2.

**Table 2:** Total treatment peripheral doses and distances from field edges.

		*Linac*		*Tomo*		
*Distance from Field Edge (cm)*	*Organ*	*Average (cGy)*	*Std Dev (%)*	*Average (cGy)*	*Std Dev (%)*	*Comparison T‐Test*
1.0	Lens of Eye ‐ Right	391.0	20.0	561.2	12.8	0.081
1.0	Lens of Eye ‐ Left	383.1	11.8	563.9	16.5	0.049
3.6	Mid‐Brain	264.1	13.1	227.4	26.8	0.310
7.4	Lung ‐ Upper Right	267.7	9.0	164.0	11.4	0.018
7.5	Lung ‐ Upper Left	257.9	12.6	168.7	10.8	0.011
16.0	Lung ‐ Lower Left	158.4	7.8	63.7	8.9	0.003
16.1	Thoracic Spine	97.2	12.3	39.3	10.5	0.006
17.7	Heart	104.2	10.4	42.8	11.8	0.003
19.9	Breast ‐ Right	77.3	4.1	32.4	14.8	0.001
20.1	Breast ‐ Left	69.7	4.4	30.2	13.6	0.001
24.2	Liver ‐ Top	59.1	5.8	26.6	12.0	0.002
25.8	Stomach	51.2	7.5	24.1	14.2	0.001
26.9	Spleen	50.8	8.4	22.7	17.4	0.002
28.0	Liver ‐ Center	46.6	5.4	23.7	15.7	0.002
36.6	Lumbar Spine	35.5	3.5	11.8	3.3	0.000
37.8	Kidneys	39.3	5.8	12.5	9.5	0.000
51.8	Ovaries	19.3	3.7	8.5	10.4	0.000
58.3	Bladder	11.2	8.3	5.9	7.7	0.001

TLD‐100s, with a useful range of 10 μGy to 10Gy, were used to measure the peripheral doses. The annealing cycle was repeated precisely throughout the study, as follows: heating the TLDs for one hour in a 400°C oven, cooling at room temperature for one half hour, heating for two hours in a 100°C oven, cooling at room temperature for eight hours. TLDs were each calibrated individually by irradiating them to known doses; readings were taken twenty‐four hours later using a Harshaw QS 3500 (Thermo Fisher Scientific, Santa Fe, MN) with a standard time‐temperature curve integrating light emitted between 50 and 300°C. The dose range used to calibrate the TLDs was determined from estimates of expected dose from the treatment planning computers. TLDs were exposed to doses of 0 cGy, 0.5 cGy, 5 cGy, and 50 cGy. The points were plotted and a calibration reading‐to‐dose four‐point curve was calculated for each TLD. These curves were checked for reproducibility. For measurement sites where the expected dose was over 50 cGy, the calibration curves were extrapolated for each TLD.

The GTV and PTV of a clinically treated base‐of‐tongue plan were fused to the phantom CT for clinically acceptable tumor delineation and nodal involvement. The treatment of the base‐of‐tongue was planned using accepted local and published practice^(^ ^6^ ^)^ and following RTOG IMRT Protocol 0522 guidelines, which allow for both linear accelerator and helical TomoTherapy‐based IMRT. In 35 fractions, the primary tumor and involved nodes (PTV70) was planned to 2 Gy per day for a total of 70 Gy and the subclinical disease sites (PTV56) was planned to 1.6 Gy per day for a total of 56 Gy. Proper coverage (100%) of the PTVs with 95% of the dose and sparing of structures was followed strictly within tolerance of the minor and major deviations. Two comparable clinically acceptable plans were produced: one utilizing Pinnacle (Philips Medical Systems, Andover, MA) 8.0 m treatment planning system for the linear accelerator based treatment, and one using TomoTherapy Hi·Art 2.2.4 system for the helical TomoTherapy plan.

**Figure 5:**
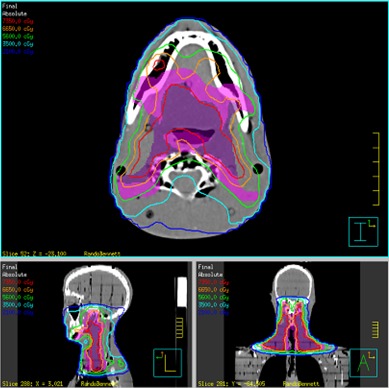
Pinnacle treatment plan.

**Figure 6:**
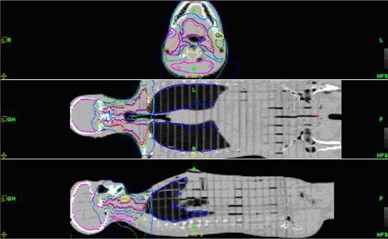
TomoTherapy treatment plan.

The linear accelerator IMRT plan, seen in Fig. 5, used nine fields which were separated into 19 split beams to accommodate the large treatment field, one field having three split beams. The 19 split beams delivered a total of 2446 MUs per fraction. The helical TomoTherapy plan (using a modulation factor of 1.5, field width of 2.5 cm, and pitch of 0.25) had a beam‐on time of 8.7 minutes and 7674 MUs per fraction. Figure 6 displays the helical TomoTherapy treatment plan. The plans were reviewed by a radiation oncologist and deemed clinical acceptability.

TLDs were loaded in the phantom and five fractions were delivered. The TLDs were read, re‐annealed, and reloaded. The process was repeated four times. Linear accelerator treatments were delivered using a Varian 2100EX (Varian Medical Systems, Palo Alto, CA), while the helical TomoTherapy plan was delivered by a TomoTherapy Hi·Art treatment system. The phantom was aligned on the 2100EX using fiducial neck markers and aligned on the TomoTherapy Hi·Art using image guidance, as is our clinical practice. The imaging dose was not subtracted from the analysis.

Systematic TLD calibration errors were reduced by distributing TLDs in different locations for each set of readings. The TLD readings were translated to dose using the calibration curves. Total treatment peripheral doses were calculated with their respective standard deviations. A Student's *t*‐test was performed on the data to determine if the data sets are statistically significantly different.

A final risk assessment was performed on the data using a method similar to Kry et al.^(^ ^7^ ^)^ based on the National Commission on Radiation Protection and Measurements (NCRP) publication No. 116.^(^ ^8^ ^)^ Each peripheral dose measurement is multiplied by its respective organ risk factor. The organ risk factors were summed yielding the total effective risk for secondary malignancy.

## III. RESULTS

**Figure 7:**
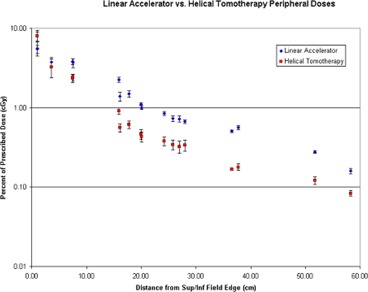
Peripheral doses as function of distance for each IMRT modality.

The TLD dose measurements, averaged and multiplied by seven, are shown in Table 2. The doses represent the total expected dose delivered over the course of 35 treatments to the respective site. Standard deviations were recorded as a percent of each average treatment dose. The data shows that helical TomoTherapy peripheral doses were on average 45% higher within one helical TomoTherapy field width, 2.5 cm, superior and inferior to the treatment field edges. Beyond 2.5 cm, the average helical TomoTherapy dose is an average of 48% lower than the linear accelerator doses, with some doses as high as 68% lower. Figure 7 shows total doses with 1σ versus distance from field edges. A Student's *t*‐test was performed using a two‐tailed, paired distribution. With a *p*‐value of 0.05, sixteen of the eighteen measurement locations were found to be statistically significantly different.

NCRP No. 116 methodology and alterations thereof by Kry et al.^(^ ^7^ ^)^ were used to calculate the risk of a fatal secondary malignancy by assigning each organ a specific fraction of the 5% per Sievert risk coefficient for a secondary malignancy. Since this estimate is valid for low dose regions up to 2–5 Sv, all of the measured peripheral doses were applicable. If multiple measurements were obtained in a given organ, the highest measurement was used for a more conservative estimate. Organ risk weighting factors were applied to the six measured organs from NCRP 116. The balance of the organ risk factors was added to the “remainder” to yield a risk‐weighted whole body dose, similar to Kry's method. The probability of a secondary malignancy, Table 3, was 5.88% for the linear accelerator based treatment and 4.08% for helical TomoTherapy. The whole body dose of 1.19 Sv for the linear accelerator treatment and 0.98 Sv for the helical TomoTherapy is the average of all peripheral dose measurement for each modality and is listed in Table 3.

**Table 3:** Secondary malignancy risk assessment of whole body doses using NCRP 116.

		*Linac*		*Tomo*	
***Specific Organ***	*%/Sv*	*Average Dose (Sv)*	*%*	*Average Dose (Sv)*	*%*
Gonads	0.10	0.19	0.02	0.08	0.01
Lung	0.85	2.68	2.28	1.69	1.43
Stomach	1.10	0.51	0.56	0.24	0.26
Bladder	0.30	0.11	0.03	0.06	0.02
Breast	0.20	0.77	0.15	0.32	0.06
Liver	0.15	0.59	0.09	0.27	0.04
**Remainder**	2.30	1.19	2.75	0.98	2.25
Lenses of Eyes					
Brain					
Spine					
Kidneys					
Spleen					
Heart					
Gonads					
Lung					
Stomach					
Bladder					
Breast					
Liver					
Bone Marrow					
Colon					
Esophagus					
Thyroid					
Skin					
Bone Surface					
**Sum**	**5.000**	Total Risk	5.88	Total Risk	4.08

## IV. CONCLUSIONS

Helical TomoTherapy treatment delivers higher doses to structures which lie within one field width of the superior and inferior field edges and lower doses beyond one field width, compared to linear accelerator IMRT. The high doses may arise due to the structure and function of the helical TomoTherapy unit. In order to deliver the full dose to the start and end of the treatment field via helical motion, the unit must start irradiation one field width above and end one field width below the actual target volume. This means that structures within one field width receive ramp up and ramp down doses. In this study, both lenses of the eyes were only a few centimeters from the superior field edge, putting them directly in the extra helical TomoTherapy field width. These doses were significantly higher, around 45%, than the linear accelerator doses.

The mid‐brain measurement point (about 3.6 cm above the field) was lower as it was beyond the extra helical TomoTherapy field width. All points beyond 2.5 cm showed helical TomoTherapy to be lower. These peripheral doses are on average less than 1% of the prescription dose. Ramsey^(^ ^2^ ^)^ reported that while the beam‐on time is 5 to 15 times longer for helical TomoTherapy, the doses were 3 to 11 times lower as the distance increases from the field edges. At 20 cm, doses of 1% for linac IMRT and 0.4% for helical TomoTherapy were measured, which agree with Ramsey's 0.4% for helical TomoTherapy. Structural design and shielding of the Hi·Art system are key factors. By contrast, this data does not agree with Mutic^(^ ^4^ ^)^ and Followill,^(^ ^5^ ^)^ who wrote that TomoTherapy would produce higher peripheral doses. Again, Mutic and Followill used the structurally different NOMOS linac‐based TomoTherapy system, in which the peripheral dose from leakage radiation should linearly follow beam‐on time.

In this study the probability of a secondary malignancy for head and neck linac IMRT and helical TomoTherapy treatments, as calculated using NCRP 116 weighting factors and an assumption of 5% per whole‐body Sievert risk, were found to be 5.88% and 4.08%, respectively. In a recent study of uncertainties in risk estimates for secondary malignancies, Kry et al.^(^ ^9^ ^)^ assert a 50% difference in dose is required to be statistically different. The risks of secondary malignancy from risk‐weighted whole‐body doses for linac IMRT and helical TomoTherapy at 1.19 Sv and 0.98 Sv, respectively, are not significantly different.

## ACKNOWLEDGEMENTS

Special acknowledgement should be given to the staff of Barrett Cancer Center at The University Hospital in Cincinnati, Ohio and Precision Radiotherapy at University Pointe in West Chester, Ohio for their assistance in this project.

The DICOM file of the segmented Rando phantom CT dataset can be obtained for future studies by request. Please contact the corresponding author for further information.
